# Impact of ultrasonication applications on color profile of foods

**DOI:** 10.1016/j.ultsonch.2022.106109

**Published:** 2022-08-01

**Authors:** Naciye Kutlu, R. Pandiselvam, Aybike Kamiloglu, Irem Saka, N.U. Sruthi, Anjineyulu Kothakota, Claudia Terezia Socol, Cristina Maria Maerescu

**Affiliations:** a Department of Food Processing, Bayburt University, Aydintepe, Bayburt 69500, Turkey; b Physiology, Biochemistry and Post-Harvest Technology Division, ICAR-Central Plantation Crops Research Institute (CPCRI), Kasaragod 671124, Kerala, India; c Department of Food Engineering, Bayburt University, Bayburt 69000, Turkey; d Department of Food Engineering, Ankara University, Ankara 06830, Turkey; e Agricultural & Food Engineering Department, Indian Institute of Technology, Kharagpur, West Bengal 721302, India; f Agro-Processing & Technology Division, CSIR-National Institute for Interdisciplinary Science and Technology (NIIST), Trivandrum 695019, Kerala, India; g Department of Genetics, University of Oradea, 410048 Oradea, Romania

**Keywords:** Ultrasonication, Anthocyanins, Betalains, Chlorophylls, Carotenoids, Food, Color

## Abstract

Food color is a feature that provides preliminary information about their preference or consumption. There are dominant pigments that determine the color of each food; the most important pigments are anthocyanins (red–purple color), chlorophylls (green color), carotenoids (yellow-orange color), and betalains (red color). These pigments can be easily affected by temperature, light, oxygen, or pH, thereby altering their properties. Therefore, while processing, it is necessary to prevent the deterioration of these pigments to the maximum possible extent. Ultrasonication, which is one of the emerging non-thermal methods, has multidimensional applications in the food industry. The present review collates information on various aspects of ultrasonication technology, its mechanism of action, influencing factors, and the competence of different ultrasonication applications (drying, irradiation, extraction, pasteurization, cooking, tempering, etc.) in preserving the color of food. It was concluded that ultrasonication treatments provide low-temperature processing at a short time, which positively influences the color properties. However, selecting optimum ultrasonic processing conditions (frequency, power, time, etc.) is crucial for each food to obtain the best color. The key challenges and limitations of the technique and possible future applications are also covered in the paper, serving as a touchstone for further research in this area.

## Highlights

• The color profile of food is a feature that provides primary information about their consumer preference.
• The present review collates information on various aspects of ultrasonication technology on color kinetics of food.
• Appropriate selection of ultrasonic processing conditions remains crucial for each food to obtain the best color.

## 1 Introduction

The color of the food is one of the most influential factors determining the consumer acceptance of foods. Color is a characteristic on which the first criticism about the product is made as it can arouse the person's appetite or turn it off [8], [75]. For instance, while purchasing red meat, the color of the meat is the first factor considered on top of other parameters. The consumer will not buy the product if the meat contains colors such as brown, green, or blue rather than red. Different names call the pigments that create the different colors. For instance, anthocyanins (blue-red), betalains (red–purple), flavonoids (yellow), carotenoids (orange, bright red, yellow), chlorophylls (green), hemoglobin (red), and myoglobin (red) are the common natural pigments that color foods [62]. One or more of these pigments can be found in foods. In addition to providing color, these pigments are also highly influential on the nutritional properties of foods, as they are bioactive compounds. Many of them have antioxidant properties and are beneficial to health [136], [62]. Color pigments can change with the light, process temperature, pH, or oxygen capacity. Therefore, the change in the color profile that occurs during food processing is quite significant.

Natural pigments are observed to transmit or reflect light on account of the selective absorption by the chromophores in a specific wavelength. Precisely, these chromophores are, in fact, responsible for the vast diversity of natural compounds that can produce color [47]. These colors are entirely associated with the structural arrangement of the chromophore comprising a molecular structure with conjugated double bonds or a metal-coordinated porphyrin. Color values in foods are commonly determined with the help of colorimeters or spectrophotometers. These values can give information about the results of chemical analysis such as anthocyanins, betalains, flavonoids, carotenoids, chlorophylls, etc. Although there are many different color scales, the most used in the food industry are the CIE (Commission International de l’Eclariage-L*, a*, b*), Hunter (L, a, b), and Munsell systems. CIE system is the most preferable among them, which consists of three-dimensional cartesian space with three mutually perpendicular coordinates that can appropriately determine the different color values in which L* is the brightness/lightness value and changes to a maximum of 100 (white) and a minimum of 0 (black). Positive a* and b* show red and yellow, negative a* and b* have shown green and blue, respectively [39]. Additionally, upon evaluating the a* b* plane polar coordinates, other major color parameters such as chroma (C*) representing vividness or dullness, and hue angle (h°), which describes the perceived color by wavelength, are also measured. Moreover, the measure of how much change the color values in foods after any treatment is determined by the total color difference (ΔE) value [139]. The coordinates and other parameters estimated from spectral measurements can be utilized to evaluate the effect of a particular treatment or processing technique on the appearance and storage stability of a product. For instance, a decrease in a* value (indicating decrement in redness) as a result of an applied process to a food material can also give information about the change in anthocyanin content which makes it very important to correctly interpret the color values in foods [138].

Non-thermal methods have been widely used in recent years as they preserve the physical and chemical properties of foods at the maximum level. With these methods, shorter processing time, higher yield, and minimal loss of nutritional properties are observed compared to thermal methods [133], [134]. The non-thermal techniques are also very substantial in preserving temperature-sensitive compounds such as taste, vitamins, color, other sensorial characteristics, etc. These methods do not involve any heat generation, although the applied process can cause a change in the temperature of the foods depending on the food characteristics [19]. For example, in a process using an electric field (such as a pulsed electric field), the temperature may increase with the movement of molecules depending on the conductivity of the food. High hydrostatic pressure, pulsed electric field, ozone, cold plasma, and ultrasonication are among the most novel non-thermal methods. With enhanced demand for high-quality foods, non-thermal processing techniques have begun to dominate different applications such as extraction [151], [163], drying [133], [198], frying [91], [51], microbial/enzyme inactivation [137], [194], pasteurization [18], [186], and thawing [185], [21] in the food industry.

Ultrasonication (US) is one of the most used non-thermal methods that aim for sustainable “green” chemistry, which employs sound waves at different frequencies for specific applications. In general, sound waves can be classified into three categories according to the frequency range: infrasonic (<16 Hz), audible (10 Hz–20 kHz), and ultrasonic waves (20 kHz-10 MHz) [176], [20], [144]). Although the discovery of ultrasonic waves dates back to the end of the 18^th^ century, their use in medicine has become widespread since the middle of the 20^th^ century. It has been applied for the first time in food technology to stabilize emulsion mixtures [92]. The ultrasonication is based on two principles; sponge or piston effect (compression-decompression) and acoustic-cavitation effect (compression-collapse). The sponge or piston is the effect that causes compression and expansion of ultrasonic waves in the medium. The cavitation, on the other hand, is an effect that causes the formation of high pressure (50–100 MPa) and high temperature (5500 °C) zones due to the release of energy by the compressing and collapsing of micro-bubbles formed in the medium during the process [14], [130], [12]. The cavitation ability of ultrasonication can change according to its characteristics (such as frequency, density), the characteristics of the medium (such as viscosity, surface tension), and external factors (such as temperature, pressure) [176], [86]. These effects have also been known to result in fractionation of the cell walls or tissues of the food materials altering different properties [27].

A study examining the different effects of ultrasonication on the color profile of foods has not been found in the literature. Accordingly, the paper delves into detail the current, up-to-date literature on color changes in foods due to different ultrasonication applications making this review the first piece in this regard. For this purpose, studies published in recent years on ultrasonication-assisted irradiation, extraction, drying, pasteurization, thawing, cooking, frying, blanching, and bleaching applications were examined together with a brief overview of different color pigments present in foods. Furthermore, the challenges encountered for all these applications are also documented to provide a clear discernment of the effect of ultrasound processing on food color.

## 2 Ultrasonication technology and color pigments

Ultrasound frequencies ranging from 20 to 1000 kHz are considered potential food processing aids owing to their ability to deliver high energy to the fluid medium. At frequencies higher than 100 kHz, lower intensities at around 1 W/cm^2^ are achieved, which are generally utilized for food quality monitoring or non-invasive diagnostic purposes where rapid, accurate, and inexpensive measures of food properties can be made during online process operations [26]. US parameters such as ultrasonic velocity, attenuation coefficient, and acoustic impedance are sensitive to intermolecular association and interactions that can be correlated to different physicochemical properties of the food materials, thus making it possible to determine any changes in the composition and structure, and physical state of food. On the other hand, applications involving food processing operations and treatments make use of sound waves with frequencies ranging from 20 to 100 kHz, often referred to as high-power or power US, generating high intensities in the range of 10 to 1000 W/cm^2^ [145]. The primary application of power US in the food industry includes destroying target cells and extracting various intracellular materials. Other prominent applications comprise modification of functional properties, emulsification, enzyme inactivation, microbial decontamination, defoaming, crystallization, freezing, drying, and concentration [14], [126], [12].

**Fig. 1:**
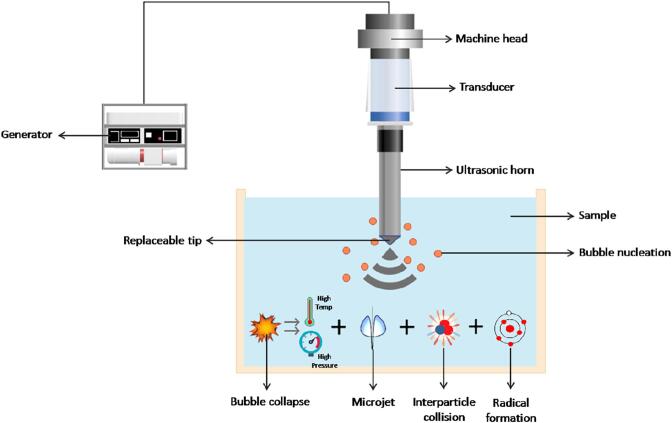
Ultrasonication working principle.

Acoustic cavitation exerts extreme physical, chemical, and even biochemical effects in the liquid medium that can modify the physicochemical properties and enhance the quality of various food systems during processing [195]. The mechanical effect of cavitation includes microjets, shear forces, shock waves, and turbulence which have applications in extraction, crystallization, emulsification, degassing, defoaming, etc. Physical effects can be summed up as increased turbulence throughout the medium primarily resulting from transient cavitation, which is explained as the catastrophic collapse of microbubbles in the fluid at lower frequencies (20 – 100 kHz) [94]. More giant bubbles are generated at lower frequencies, which cause more violent cavitation collapse (Fig. 1), leading to higher localized temperatures and pressures [95]. However, with increased frequency, the number of collapses increases with unit time, leading to a more uniform and less intense acoustic field. The lower frequency is favored for applications where the generated OH radicals might adversely influence the integrity of food constituents since a more significant number of radicals get generated with the increase in the number of active bubbles at a higher frequency. The microscale implosion can generate higher temperatures in the range of 2000 to 5000 K and pressure ranging from 30,000 to 120000 kPa for a short time, depending on the frequency employed [145]. The process can also enhance the heat and mass transfer in different ways using high-velocity interparticle collisions, accelerating eddy diffusion, and internally distributing perturbing particles of the materials into the medium. At intermediate frequencies (200 – 500 kHz), with the better generation of active bubbles, the chemical effects become dominant with less frequent transient cavitation events. The high temperature also generated results in the direct formation of primary radicals such as H and OH, depending on the number of active bubbles generated and the bubble temperature [94].

The influence of US power and sonication time is another major topic of discussion as far as the efficiency of the process is concerned. Higher power induces greater shear forces that can cause significant modifications in the food materials that find it necessary to optimize the input power to achieve target results at minimum power. Proper US frequency to be used in specific applications is usually correlated with the input power, as high amplitude and low frequency (20–100 kHz) are associated with high acoustic power, whereas low amplitude and high frequency (100–1000 kHz) are linked to low US power [63]. The longer the sonication time required for treatment, the faster the sonication device will degrade. Therefore, for applications involving longer treatment times, lesser amplitudes are recommended to prevent abrupt corrosion of the ultrasonic transducers [28]. Additionally, it is also established that US, when applied in combination with temperature and pressure, the process being called thermosonication and manosonication, respectively, have demonstrated to produce a synergistic effect enhancing the efficacy of the sonication process [36]. The basis of US processing is the equipment used, i.e., the sonoreactor, design, and size. The transducer plays another significant role in the sonication process, with the piezoelectric systems being more efficient than the magnetostrictive system. However, the latter can generate high levels of acoustic power intensities [25]. Therefore, optimization of operating conditions before the application is recommended to avoid adverse effects of US on food.

High power US can be applied using sonication baths or ultrasonic immersion probes with different lengths, diameters, and tip geometries, depending on the applications. A flat bottom vessel with minimum thickness is preferred for ultrasonic baths to attain minimum waves’ reflection. However, baths are less preferred in chemical applications because of the lower reproducibility of the reactions obtained. Also, the intensity delivered is low and is very much attenuated by the water in the bath and the glassware walls used for the experiment [27]. In the case of probes, less attenuation happens because of the direct immersion of the probe into the reaction flask. Regarding the shape and diameter of the probe, it was ascertained that stepped probes gave the highest amplitude magnification and exponential probe shape with a small diameter at the contact surface makes them suitable for micro-applications.

The color of food plays one of the most critical roles in deciding the consumer perception of any processed or unprocessed food, making it an essential parameter that is given significance in the food industry. With a rising inclination towards health concerns, incorporating natural pigments as substitutes for their synthetic counterparts is the current marketing trend in various industrial fields. Pigments are molecules emitting color, usually derived from plants, animals, insects, and microbes, with plants being the primary source [10]. Over the past years, with the growing popularity of these compounds owing to their biodegradable, renewable, non-toxic, and environmentally friendly characteristics, bounding research has been done to investigate their occurrence and levels in different foods along with the factors that influence the composition.

Pigments present in foods, especially in fruits and vegetables, belong to diverse groups of chemical substances which differ in color, stability, solubility, and sensitivity to environmental conditions in the presence of other substances [130]. Mainly, pigments obtained from plant origin can be grouped into four main groups, namely, the green chlorophylls (E 140), the yellow/orange/red carotenoids (E 160a), the red/blue/purple/pink anthocyanins (E 163), and the red betanins (E 162) [121]. The chlorophylls represent a group of porphyrins; carotenoids belong to a group of isoprenoid derivatives, anthocyanins are included in the group of phenolics, whereas betanins do not belong to a specific group. Based on their chemical structure, natural pigments can be again classified as tetrapyrroles, tetraterpenoids, flavonoids, anthraquinones, and betains [9].

## 3 Ultrasonication applications on color profile of foods

As discussed, ultrasonic processing has been successfully demonstrated for different food processing and preservation applications. With color being an important trait of other food products, the influence of US on the color profile of food is an imperative topic to be discussed for expanding its application window to further boundaries. For that reason, the effect of different ultrasonic applications on the color of some foods is summarized in Table 1. The following sections deal with significant ultrasonication applications and their impact on important color parameters of several foods in more detail.

**Table 1:** Recent selected studies about the effects of ultrasonication on food color.

Food material	Application	Performed conditions	Results	References
Red wines	US assisted irradiation	US power: 950 W Time: 14 and 28 min Frequency: 20 kHz US device: Ultrasonic probe	Increased the color intensity and visual properties of the red wine with ultrasonic treatment Under suitable ultrasonic irradiation conditions, it could be speeded up some aging reactions and thus shorten the winemaking time	Zhang and Wang [196]
Goat milk		US power: 150, 200, 300 and 400 W Time: 15 s Frequency: 20 kHz Temperature72 °C US device: Ultrasonic probe	The color values seem to increase with ultrasonication compared to raw milk, but no statistically significant change has been observed between the valuesIt has shown that the ultrasonication irradiation did not initiate Maillard reactions (relevant to a* value due to redness)	Ragab et al. [143]
Beetroot		US power: 300 W Time: 10 and 15 min Frequency: 21 kHz US device: Ultrasonic bath	The pretreatment time of beetroot from 10 min to 15 min did not cause a change in the color values Inbeetroot powder,an increase in the red and yellow color values was observed with the increase of the time It has been reported that the most positive effect on color values was thanks to the 15-min ultrasonic irradiation	Janiszewska-Turak et al. (2021)
Plum juice		1. (US)-US power: 600 W Time: 30 min Frequency: 38 kHz Temperature: 45 °C2. (UEZ15) -US power: 600 W Time: 15 min Frequency: 38 kHz Temperature: 45 °C Enzyme concentration, temperature and time: 0.2 % (v/v) at 90 °C for 3 min3. (UEZ30) -US power: 600 W Time: 30 min Frequency: 38 kHz Temperature: 45 °C Enzyme concentration, temperature and time: 0.2 % (v/v) at 90 °C for 3 min4. (UEZ60) -US power: 600 W Time: 60 min Frequency: 38 kHz Temperature: 45 °C Enzyme concentration, temperature and time: 0.2 % (v/v) at 90 °C for 3 min5. (EZ) -Enzyme concentration, temperature and time: 0.2 % (v/v), at 45 °C for 60 min US device: Ultrasonic bath	The brightness of juice increased in UEZ and EZ juice samples, while their redness and yellowness were significantly lowered Changes in the redness of plum juice could also be related to enzymatic browning By UEZ and EZ, a* and b* values were significantly decreased compared to US and control It was seen that it showed a positive correlation with the color values The UEZ15 was the most appropriate pretreatment conditions	Olawuyi et al. [131]
Quinoa, amaranth and millet	US assisted extraction	US power: 150 W Time: 7 min Frequency: 37 kHz Temperature: 50 °CProtease enzyme (2. Pretreatment) US device: Ultrasonic bath	The lowest ΔE for quinoa and millet was obtained by the enzymatic-assisted ultrasonic method, while the lowest ΔE for amaranth was acquired when the ultrasonic method was used alone	Kurek et al. [99]
Strawberry juice		US power: 400 W Time: 4, 8, 12 and 16 min Frequency: 20 kHz US device: Ultrasonic probe	There was a slight increase in L* value up to 12 min and a decrease in the 16. min with the ultrasonic application An increasing trend was seen in the ΔE with increasing time The time of 12 min (ΔE: 0.42) is beneficial for preserving the color properties	Wang et al. [180]
Hemp seeds		US power: 800 W Time: 30, 60 and 90 min Frequency: 5 kHz Temperature: 30, 45 and 60 °C US device: Ultrasonic bath	The green color is due to its high content of chlorophyll and carotenoid pigments The effect of temperature on the color index was found to be linear, while extraction time was quadratic effects The optimum conditions were determined as 54.40 min for the extraction time and 40.26 °C for the temperature	Kenari and Dehghan [93]
Cornelian cherry		1. (M)- Time: 24 h Temperature: 25 °C2. (UA) - US power: 320 W Time: 1, 20, 40, 60, 80 min Frequency: 35 kHz3. (OHAU) -Electric field: 20, 30 and 40 V/cmTime (holding) : 1, 10 and 20 minTime (sonication) : 1, 20, 40, 60 80 min US power: 320 W Frequency: 35 kHz US device: Ultrasonic bath	The lightness (L*) value of the extract obtained by OHAU extraction at the optimum point was lower than the value obtained by the UA and MThe redness value (+a*) was found to be highest in OHAU conditions The highest total phenolic compound was obtained by OHAU extraction and the color values supported	Kutlu et al. [100]
Common carp	US assisted thawing	US power: 0, 100, 300 and 500 W Frequency: 30 kHz US device: Ultrasonic tank	300 W ultrasonic power was the best method to keep the original color	Sun et al. [171]
White yak meat		US power: 200, 400 and 600 W Frequency: 20 kHz US device: Ultrasonic tank	The ultrasonic treatment increased L* and b* values	Guo et al. [65]
Pork meat		US power: 150 W Frequency: 40 kHz US device: Sonicator	The a* and b* values of the samples thawed by ultrasonic in brine were significantly lower than the control group, whereas the ΔE values were significantly higher The thawing treatment by ultrasonic in water did not show any differences in the a*, b*, and ΔE values from the control	Hong et al. [71]
Bighead carps		US intensity: 0.135 W/mL Frequency: 28 kHz US device: Ultrasonic bath	There was no significant difference in the a* and b* values in terms of thawing methods, and the application of ultrasonic did not affect the color change in fish	Li et al. [109]
Mango pulp		US intensity: 0.037, 0.074 and 0.123 W/mL Frequency: 28 kHz US device: Ultrasonic bath	The color of mango pulp was not detrimentally affected by ultrasonic	Liu et al. [113]
Blueberries		US intensity: 20 W/g Time: 20 min Frequency: 45 kHz US device: Ultrasonic bath	The L*, a*, and b* values of the blueberries increased with ultrasonic treatment, and this process had a significant influence on the color of the fruit	Chen et al. [30]
Red radish		US power: 120, 180, 240 and 300 W Frequency: 20 kHz US device: Ultrasonic tank	The L*, a*, and b* values of all thawed samples significantly decreased (P < 0.05) compared to the fresh sample The ultrasonic treatment better preserved the redness of the red radish, and the lowest ΔE values belonged to the samples thawed by ultrasonic	Xu et al. [190]
Mushroom		1. US intensity: 16.65 W/kg Frequency: 28 kHz 2. US power: 150, 250 and 350 W Frequency: 20 kHz US device: Ultrasonic-probe	The utilization of ultrasonic bath and probe in the power of 250 W and 350 W improved the color and color index of the thawed samples	Farahnak et al. [54]
Mortadella	US assisted cooking	US power: 301 W Time: 450 and 900 s Frequency: 25 kHz US device: Ultrasonic bath	There were no significant differences in the color of the ultrasonic-cooked mortadellas In the study, a*/b* values of the ultrasonic-cooked samples were similar to the control sample	da Silva et al. [44]
Pasta		US power: 50 W Time: 8 min Frequency: 40 kHz US device: Ultrasonic-probe	Ultrasonic cooking better preserved the yellow color in the final product, indicating less pigment loss	RasoPueyo and Álvarez Lanzarote (2018)
Corn		US power: 1350 W Time: 1 h Frequency: 20 kHz US device: Ultrasonic probe	The ultrasonic treatment made significant differences between the L*, C* and ΔE values of the samples	Janve et al. [82]
Aquafaba		US intensity: 34 and 67 W/cm^2^ Time: 10, 20 and 30 min Frequency: 20 kHz US device: Ultrasonic probe	The ultrasonic application at the highest intensity for 30 min. increased the stability, texture, and color of foams	Meurer et al. [119]
Brown seaweed	US assisted drying	US intensity: 7, 35.61 and 75.78 W/cm^2^ Time: 10 min Frequency: 20 kHz US device: Ultrasonic probe	After rehydration L* values were increased according to controlThe lowest ultrasonic power (7 W/cm^2^) gave the highest increase in L* value	Kadam et al. [87]
Tomato slices		US power: 300 W and 13 L Time: 0, 20 and 40 min Frequency: 25 kHz US device: Ultrasonic bath	L*, a* values increased with an increase in the ultrasonic treatment 40 min ultrasonic treatment effect color properties ΔE values were better with 20 min US treatment	Horuz et al. [72]
Apple		Frequency: 40 kHz US device: Ultrasonic dryer	L* value of US dried samples was closed to fresh samples a* and b* values were increased ΔE values of ultrasonic dried samples were smaller than hot air-dried samples	Kahraman et al. [88]
Rainbow trout fillet		Frequency: 40 kHz US device: Ultrasonic bath with vacuum pump	L* values of ultrasonic assisted vacuum dried samples were higher than hot air-dried samples Low a/b ratios were provided with ultrasonic assisted vacuum drying	Ismail and Kocabay [78]
Araçá-boi pulp	US assisted pasteurization	US power: 300 W US intensity: 1000, 3000, 5000 and 7000 J/g Frequency: 19 kHz US device: Ultrasonic probe	Ultrasonic treatment affected pulp color properties 5000 and 7000 Ultrasonic treatment was increased L* value of pulp 1000 J/g ultrasonic treatment increased Hue angle	de Araujo et al. [46]
Raw milk		US power: 100 and 457 W Frequency: 19 kHz US device: Ultrasonic probe	High energy densities increased L* value and decreased h values. 457 W ultrasonic treatment provided 3.9 log aerobic mesophilic heterotrophic bacteria inhibition	Scudino et al. [157]
Strawberry juice		Time: 5, 10 and 15 min Frequency: 40 kHz Temperature: 25, 40 and 50 °C US device: Ultrasonic bath	50 °C 15 min ultrasonic treatment provided 2.92 cfu/ml log aerobic mesophilic bacteria, 2.05 cfu/ml log mold and 2.86 cfu/ml log coliform (35 °C) inhibition The highest L* value was detected in 50 °C 15 min ultrasonic treatment Color parameters were less affected with 25 °C treatment (smallest ΔE value) Hue angle increased with time and temperature	Menelli et al. [118]
Perilla leaves	US assisted blanching	US power: 360 W Time: 1, 2 and 3 min Frequency: 40 kHz Temperature: 70 and 80 °C	Ultrasonic treatment increased greenness (lower Δ*E* and lower a*) L* and b* values decreased by heat 70 °C,3 min ultrasonically treated samples gave the lowest Δ*E* and a* values	Song et al. [167]
Mushroom	US assisted frying	US power: 120 W Frequency: 28 kHz US device: Ultrasonic source	L* values were highest in UMVF Lowest a* value provided by UMVF b* value of UMVF was higher than other methods	Devi et al. [49]

### 3.1 Effect of ultrasonic irradiation

Ultrasonic irradiation is commonly used as a pretreatment or treatment in food processing. The principle of this process is mainly based on the acoustic cavitation phenomenon, which is the primary mechanism of ultrasonication [130]. As mentioned in the previous sections, the exceptionally high temperature and pressure resulting from acoustic cavitation can break down water molecules into free chemical radicals, and these radicals can speed up specific chemical reactions. This may facilitate the fragmentation of plant cells [173]. The most critical factors determining the effect of ultrasonic irradiation are temperature, pressure, ultrasonic frequency, and ultrasonic power [90].

Ultrasonic irradiation is used in many kinds of foods for different purposes. For example, in foods rich in enzymes such as polyphenol oxidase or peroxidase (such as potato, quince, apple, etc.), ultrasonic irradiation can prevent quick browning after cutting [120], [135]. Ultrasonic irradiation treatment/pre-treatment application also ensures increased effectiveness of the succeeding process or unit operation to be applied, for instance, drying or cooking. In addition, it is widely used to increase the extraction of bioactive compounds from fruit/vegetables and all kinds of food by-products [128], oil recovery from oil seeds plant [85], bioethanol yield from lignocellulosic materials [90], or alcoholic/nonalcoholic beverages processing [114], [35]. Besides these, using ultrasonic irradiation alone can also significantly affect foods' physical and chemical properties [197]. For this reason, it is an application that often finds utilization in the food industry.

Color, as mentioned before, is an indicator of the aesthetic value and acceptability of food products. US irradiation has increased many food products' color intensity and visual appearance. This could be attributed to the instant high temperatures and pressures generated locally due to acoustic cavitation, which could initiate specific chemical reactions that could have accelerated the formation of some stable pigments. For example, Zhang and Wang [196] for red wines and Fan et al. [53] for cucumber showed that US irradiation positively affects color. In a different study, Calderón-Martínez et al. [23] obtained an increase in luminosity (L*) value upon ultrasonic irradiation of gulupa (Passiflora edulis Sims) pulp which was attributed to the decrease in particle size owing to cavitation, which increases light reflection, luminosity, and carotenoid degradation. This was also explained by the partial precipitation of unstable particles in the pulp because of the rise in temperature at the microsites during US treatment. The US can also result in the isomerization of carotenoids present in the gulupa pulp, which increases the redness and yellowness of the pulp by forming compounds with brown color properties, resembling those occurring during the browning process.

Although the temperature is the main factor in the formation of the Maillard reaction, it is known that US radiation does not trigger this reaction due to its short processing time. As evidence for this case, in the study conducted with goat milk, it was emphasized that US irradiation did not initiate the Maillard reaction. Therefore the color values of raw milk and treated milk were not statistically different [143]. In a more recent study, Comarella et al. [35] noticed an increased concentration of monomeric anthocyanins in “Isabella” grape juice due to US irradiation because of the synergism between sonication time and temperature leading to increased release of bioactive compounds. They divulged that the free radical formation and mechanical perturbation could trigger the defense responses in plants activating secondary metabolism and stimulating the production of phenolic substances like anthocyanins and flavonoids, which could, in turn, improve the color properties of such products.

Undesirable color changes can be prevented since US radiation causes the polyphenol oxidase enzyme to be inactive. For example, in US irradiation-treated prune juice, Olawuyi et al. [131] observed decreased polyphenol oxidase activity, which is associated with enzymatic browning. Moreover, the improved color was also associated with the increased release of anthocyanin from the fruit skin into juice. In brief, ultrasonic irradiation can be regarded as an effective tool for positively influencing the color values of food under short treatment times without altering the important quality aspects. US irradiation was also found to reduce the activities of specific enzymes responsible for initiating browning, thus preserving the natural color of the food.

### 3.2 Effect of ultrasonic extraction

Ultrasonication improves heat and mass transfer by collapsing cell walls using the cavitation effect and has recently been used as a stand-alone process or as part of a multi-staged procedure for the extraction of bioactive compounds (such as phenolic compounds, anthocyanins, carotenoids, flavonoids, antioxidants, etc.) from foods. Ultrasonic assisted extraction (UAE) has many advantages, such as providing greater solvent penetration into the food material, high product yield, high reproducibility, low solvent consumption, and extraction of temperature-sensitive compounds. In addition, it can be easily used in integration with other extraction methods [15]. Many researchers have reported that the extraction yield (especially bioactive compounds) with UAE is higher than traditional methods [178], [17], [100].

Plenty of color pigments (such as anthocyanin, carotenoid, betalain, chlorophyll, etc.) are used as natural colorants to produce processed foods in the food industry. For this reason, it is of great importance to obtain the color materials with the most effective extraction method (in terms of the highest yield). When the effect of ultrasonication on color pigments was examined, it was seen that the most significant impact was for anthocyanins. It has been reported that the yields of anthocyanins, which are high in red fruits, are higher in UAE compared to traditional extraction methods such as maceration, water-bath, or solvent extraction [2], [100]. In addition, the ultrasonication effect is of major significance in getting betalain from purple-colored foods such as beetroot [59], in obtaining chlorophyll from green microalga (*Chlorella vulgaris*) [97], or in getting carotenoids from fresh and dried carrot tissues [129]. The most substantial factors affecting the color profile during ultrasonic extraction are ultrasonic power, frequency, light, sonication time, solid/solvent ratio, solvent type, and temperature.

It is seen that UAE increases the yield of natural colorants (carotenoids, phenolic compounds, anthocyanins, or chlorophyll) from plants compared to other commercially practiced methods, the primary reason being increased mass transfer rates a result of acoustic cavitation [166]. For example, Fernández-Ronco et al. [57] observed reduced degradation of carotenoid pigments with UAE while extracting oleoresin from *Capsicum annuum* by dint of shorter exposure times to higher temperatures*.* They found a direct influence of carotenoids on color capacity, thus ensuring a stable color profile upon US-assisted extraction. Similar results have been observed that the phenolic compounds of Vitis vinifera Cabernet Sauvignon [45], jussara (*Euterpe edulis* M.), and blueberry (*Vaccinium myrtillus*) [150], anthocyanins of bayberry juice [24] and bilberry [177], and curcumin of turmeric rhizomes (*Curcuma longa* L.) [127]. In conclusion, based on all these studies, it has been observed that US extraction facilitates the extraction of bioactive compounds adjacent to the plant cell wall and increases the yield.

When the effect of US extraction conditions (sonication time, solid to solvent ratio or ultrasonic power, etc.) on the color was examined, it was seen that different trends were obtained depending on the changing food materials. For example, Rocha et al. [150] reported that the sonication time did not affect the color values, but the effect of the fruit to solvent ratio was significant. In other respects, it has been reported that an increase in L value, in other words, a darkening in color, was observed because of higher phenolic compound yield depending on a rise in sonication time and the solvent amount. Also, with longer sonication times, degradation of the chlorophyll pigment was reported, which provides the green color [96]. Similarly, it has also been reported that the ultrasonic effect can cause cell disruption leading to the release of intracellular compounds that may affect product color due to the long sonication time [180]. In a study, Varo et al. [177] examined the effect of ultrasonic power applied in extraction; it was reported that as the power increased, the L* values of bilberry juice’s extracts decreased, and the a* values increased. It was also observed that the anthocyanin content decreased contrary to the a* value with increasing ultrasonic power. This finding has been explained by the formation of different compounds (such as flavanols) that provide the red color, apart from anthocyanins. In addition, it has been stated that anthocyanins might react with different polymers that help the formation of red color, causing this color value to increase. On the contrary, da Rocha and Noreña [40], in their study to obtain anthocyanin from grape pomace by US extraction, reported that anthocyanin yield increased with increasing sonication time, and L* (reduced) and a* (increased) values changed in parallel. The reason for this finding has been explained by the increase in temperature with increasing sonication time and ultrasonic power, and this increment in temperature has supported the extraction yield.

UAE is also an effective method for color preservation of oil compared to other traditional techniques. And many studies to support this finding are available in the literature. For instance, Samaram et al. [154] used the UAE method to extract the oil from the seeds of Malaysian Sekaki papaya fruit, and the results were compared with solvent extraction (SE). It has been observed that the color of the oil changes according to the carotenoids and chlorophyll content. The redness (+a*) and yellowness (+b*) values of the oil obtained by UAE were found to be lower than the values of SE, and it was indicated to be due to the much shorter extraction time. In conclusion, the UAE method was found to be suitable, and it was stated that the production of the light color oil was an indicator of quality. Tan et al. [172] also found a similar result, who produced virgin avocado oil using US extraction. In addition, in an experiment to obtain pectin from grapefruit peel, it was found that the color of pectin is negatively affected by high temperatures and long time, which suggests the application of ultrasonication for getting a better color profile in comparison to the traditional extraction [179]. In another study on oil extraction from hemp seeds by ultrasonic extraction, the color indexes of the oil samples at the end of the extraction were examined. The green color of hemp (*Cannabis sativa* L.) seed oil is due to its high chlorophyll and carotenoid pigments. It was seen that the color index decreased (more yellow color) when the solvent was hexane, and the color index increased (greener color) because more chlorophyll was extracted as the isopropanol ratio increased (maximum yield in organic solvent) [93].

A study comparing ultrasonic probe and bath was found in the literature, and the results were quite remarkable. Guava pulp (GP) and guava waste (GW) were used as materials for extraction in this study [111]. The color values of the obtained extracts by using maceration, ultrasonic bath, and ultrasonic probe were compared. In conclusion, it was observed that GP and GW extracts obtained by ultrasonic bath had the most attractive color, with the highest a* and b*. Color results were consistent with the carotenoid concentration of the extracts. It has been reported that the extracts obtained by the ultrasonic probe were the least attractive color because of their low concentrations of lycopene and β-carotene.

In conclusion, ultrasonic-assisted extraction generally improves the color characteristics of food materials compared to traditional extraction methods. Some of the most important parameters affecting the efficiency of this method are ultrasonic power and sonication time. In addition to these, the frequency and applied temperature of the used ultrasonic probe or bath are also critical factors. Recently, better color values have been obtained as a result of using ultrasonic extraction in combination with different extraction methods, and it has also been proven by many researchers in the literature [99], [67], [116], [159]. When all these parameters/combinations are determined correctly, it is a proven fact that the method has a protective effect on color pigments.

### 3.3 Effect of ultrasonic thawing

The availability of ultrasound technology in the food industry has been widely investigated for its applicability in operations such as thawing, drying, cooking, freezing, drying/dehydration, brining, etc., because of its reduced energy consumption, improved processing efficiency, and safety and quality of products. The application of US assists or accelerates traditional methods so that the processes can be done rapidly or efficiently. Thus, the disadvantages of traditional techniques are eliminated or improved [173].

Thawing is one of the most important stages for frozen products because the final quality of these products depends on the thawing process. Thawing the bulky frozen products takes much more time than freezing [173]. This can cause costly consequences for industrial food processing and make it challenging for small and large-scale caterers [141]. In addition, products are exposed to microorganisms or physical and chemical changes during thawing. Besides, a rapid increase in the temperature can be seen on the surface of frozen products compared to the interior, and this may cause damage to the surface of a material subject to thawing. For this reason, various technologies are utilized to increase the thawing rate, make the process faster, and encourage heat generation in the food [173].

It was reported that thawing conditions negatively affected the quality of the products when it was not appropriate [61], [81]. Ascorbic acid degradation [70], decrease in antioxidant activity, and carotenoid degradation [168] were possible changes in the product. Therefore, it is important to determine suitable conditions and methods for thawing. Recently, novel thawing methods were developed, such as high-pressure, microwave, or US thawing [84]. US treatment has many advantages compared to traditional methods due to the process's rapid and stable nature [161]. The micro-steaming induced by US could increase the heat and mass transfer, thus reducing the resistance of this transfer phenomenon at the ice/liquid interface [32]. In addition, data from several studies suggest that the US process can reduce thawing times. Cheng et al. [31] and Gambuteanu and Alexe [61] determined that the thawing times for frozen edamame and pork thawed by US were reduced by 54.39 % and 87 %, respectively. However, the findings from some studies have suggested that US treatment causes some quality problems (carotene and ascorbic acid degradation, and dark color) in fruit juice or nectar [80], [140]. In addition, localized heating, surface over-heating, high power requirement, and poor penetration are other disadvantages [31], [21], [110]. Therefore, researchers have shown an increased interest in determining the best US-assisted food thawing process [142].

In a study using blueberry, Yuan et al. [193] stated that freeze-ultrasonic thawing technology was an effective method for extracting anthocyanins and other plant pigments. The high temperature and US frequency can damage the structure of anthocyanins. However, repeated freezing– ultrasonic thawing cycles can be a feasible method for this purpose. In the study, while the color of the fresh fruit peels was intense purple-red, the colors of the frozen and then thawed samples were significantly lighter. In other words, the density of the peel color is lower in the samples with US treatment. A considerable amount of literature has been published on the US-assisted food thawing process. Products such as edamame [31], cooked potato flour [160], mango pulp [113], white yak meat [64], pork [60], [61], [105], [182], Peru squid [107], fish products [21], [108], [161], [29], [171], chicken breast [199] were used in these studies.

Pigment degradation and lipid oxidation can be considered among the causes of color changes in meat [188]. Sun et al. [171] investigated the effect of US -assisted thawing on the common carp (*Cyprinus carpio*) and its muscle quality. The researchers determined that the 300 W ultrasonic power was the best method to keep the original color of the sample. The L* value of the control samples was the lowest among all samples. In the US treatment, the increase in US power first reduced the L* value and then increased. The reason for this may be that the muscle structure was damaged, and some of the free water migrated to the surface, increasing the L* value of the sample. A study determined that the structural characteristics of myosin were improved by US-assisted thawing when the suitable US power was chosen [64]. In addition, this treatment increased the myosin and sulfhydryl content and improved the water holding capacity of the muscle protein. This may explain the lower L* value of the sample thawed by US at 300 W. However, the high US power (500 W) increased the L* value and devastated the muscle structure [162]. The thawed sample had a lower a* value than the control samples. There are two likely causes for this situation: (i) the water loss along with the loss of a part of the pigment during thawing and (ii) the oxidation reaction during thawing. In addition, the utilization of higher US power can lead to the oxidation of muscle pigments, resulting in a decrease in the a* value and an increase in the L* and b* values [171]. There was also an increase in the b* value of the sample thawed by US in the same study.

Guo et al. [64] determined the L*, a*, and b* values of the white yak meat thawed by US. The control and sample treated with US at 600 W had the highest L* and b* values. These were higher than those of the sample thawed by US at 400 W by about 17 % and 27 %, respectively. However, the sample treated with US at 400 W had the highest a* value. The same sample showed a decrease in L* value compared to the control, and it had a lower and consistent b* value. After thawing, the intension in the L* value of the meat was deleterious due to the spread of free water to the surface. US technology affects a* value in meat because the production of free radicals may make heme pigments unstable due to promoting the oxidation, and the treatment may also change the chemical properties of myoglobin and hemoglobin owing to the thermal and acoustic effects of the US [74]. In addition, the increase in the b* value of meat is mainly due to lipid oxidation [64]. A recent paper by Wang et al. [182] investigated the effect of different thawing methods on gelling properties of protein from porcine meat. They determined the whiteness value of myofibrillar protein gel from fresh meat samples as 76.50. This value decreased by 0.40 % after US treatment, and this decrease was not found significant (p > 0.05). However, the decline in whiteness values of myofibrillar protein gel from thawed by US may be related to the low water holding capacity of the same samples. For the reason that there was a positive correlation between these values and the low water content could make the color of the gel darker [106]. In another study, Hong et al. [71] evaluated the qualities of pork thawed by US and immersion either in brine or water. The pork samples became discolored by the application of US in brine. None of the treatments differed from the control in L* values (p > 0.05). The a* and b* values of the samples thawed by US in brine were significantly lower than the control group, whereas the ΔE values were significantly higher (p < 0.05). However, thawing treatment by US in water did not show any differences in the a*, b*, and ΔE values from the control (p > 0.05). Gambuteanu and Alexe [61] stated that the US-assisted thawing process could preserve the quality of frozen products and reduce the thawing time.

Li et al. [109] studied bighead carps and showed that the L* values of the thawed samples were higher than the control after freezing and thawing treatments (p < 0.05). However, there were no significant differences between the a* values of the frozen-thawed samples regardless of the thawing treatments (p > 0.05). In contrast, these values were significantly lower than the control (p < 0.05). The b* values of the treated samples were significantly higher than the control (p < 0.05), but the researchers reported no significant difference in these values in terms of thawing methods. The study also indicated that the application of US did not affect the color change in fish and inhibited lipid oxidation. These results seem to be consistent with another study conducted by Cai et al. [22]. They found that the a* values of thawed largemouth black bass (*Micropterus salmoides*) were significantly (p < 0.05) lower than that of the fresh sample, and the b* values of thawed samples were significantly higher than that of the fresh sample. However, US-assisted thawing significantly reduced the L* values of thawed samples compared to fresh samples (p < 0.05). On the other hand, an opposite situation was observed in the ΔE* values due to protein aggregation during thawing. In these studies, the researchers stated that the water content and distribution affected the color of the fish samples. Thus, it was expressed that the higher L* values indicating surplus moisture on the surface could be the reason for the higher L* values. In addition, the lower a* values were mainly associated with myoglobin oxidation, while the higher b* values were closely related to lipid oxidation [22], [109]. In a recent study, Wang et al. [183] examined the effects of US on the thawing of quick-frozen small yellow croakers. They found that the color characteristics (L*, a*, b*, ΔE values) of croackers thawed by US were not significantly different from the fresh sample. However, the redox stability of myoglobin was negatively affected due to the increase of glycolytic enzymes [55]. This may be the reason for the color stability of the thawed samples by US was higher than the water-thawed samples. The researchers stated that the amount of glycolytic enzymes in the thawed samples by US was lower than that in water-thawed samples. Therefore, the thawing process by US may be more appropriate for the color stability and quality of the croackers.

Thawing by US is also widely used in fruit and vegetable products. Liu et al. [113] used the US technique during the thawing process of mango pulp in their study. In the sensory evaluation, the color of all thawed samples by different US intensities did not show a significant difference (p > 0.05); that is, the color of mango pulp was not detrimentally affected by US. The US application slightly affected the color of mango pulp, and it could be related to the browning reaction as indicated by 5-hydroxymethylfurfural (HMF). It means there was no significant difference in color change values of thawed samples by US. In contrast, Chen et al. [30] determined that the blueberries’ L*, a*, and b* values increased with US treatment, and this process had a significant influence on the color of the fruit. The blueberries kept the original color except for the treated fruits by US. This could be attributed to the loss of anthocyanins during the US process. In addition, cavitation-induced damage was noted to be responsible for such a change [165].

Another study examined the effects of US-assisted thawing on the quality of edamames frozen using different freezing methods [31]. The thawing treatments at 600 W, 900 W, and 1200 W made no significant difference in the treated samples’ L*, a*, and b* values. However, the a* and b* values of the thawed samples by US were statistically higher than the control, whereas the L* values were lower (p < 0.05). Cheng et al. [31] found considerable differences (p < 0.05) between ΔE values of the samples after the thawing process. The thawed samples using US power at 900 W had the lowest ΔE values, which indicated that the original color of the sample was better maintained. Apart from these, chlorophyll is an important pigment in green vegetables and fruits, which is converted into pheophytins during freezing and thawing. This causes the color of green vegetables to change from green to brown. Chlorophyll contents of edamame samples thawed using US power at 600 W, 900 W, and 1200 W were significantly higher than samples thawed using a 300 W power level (p < 0.05).

A US-assisted water thawing process was also applied to red radishes [190]. It was indicated that the low-frequency US was an efficient method to ease the thawing process and maintain the product's structure, vitamin C, and color. Different thawing methods were also used in the study, and the L*, a*, and b* values of all thawed samples significantly decreased (p < 0.05) compared to the fresh sample. The US treatment better preserved the redness of the red radish, and the lowest ΔE values belonged to the samples thawed by US. Accordingly, the improvement in the color characteristic of red radish was noted during the thawing process assisted by the low-frequency US. Farahnak et al. [54] used a different material in US thawing at high power levels. They stated that the best effect on mushroom color index and textural properties was obtained when the high US power (350 W) was used. The samples thawed by US at 150 W had the lowest L* and whiteness values but the highest ΔE values. However, the thawed samples using US power of 350 W had the highest whiteness but the lowest ΔE values. These findings suggested that, in general, the utilization of ultrasonic bath and probe in the power of 250 W and 350 W improved the color and color index of the thawed samples. According to Lagnika et al. [101], the high US power could inactive the enzymes such as polyphenol oxidase and so decrease the browning reactions. For this reason, the higher whiteness of the thawed samples at 350 W could be related to this situation. As mentioned before, another possible explanation is that the higher water holding capacity of the samples thawed by US at 250 W and 350 W could result in the higher whiteness of these samples [182].

As a result, the US treatment applied during thawing affected the color values of the samples differently. Different materials and processing conditions may be the reason for these contradictory results. However, this method is still recommended in thawing processes in terms of both its short duration and various benefits, although it has negative effects on some products.

### 3.4 Effect of ultrasonic cooking

In traditional cooking methods, the interior of the food may not be fully cooked while the surface is overcooked, which causes a loss in product quality. The application of US during cooking may offer potential advantages due to its ability to improve mass and energy transfer characteristics [27], [7]. It is claimed that US cooking reduces energy consumption. For instance, compared to traditional cooking, US cooking was found to be better in beef in terms of cooking speed, energy efficiency, and moisture retention [27]. This method is recommended as a new and rapid method because it is stated that it can improve the textural properties of cooked meat [7]. The panelists noted much better myofibrillar tenderness in US-cooked meats. The high-intensity US can increase the water-binding ability of meats, which can explain these changes in moisture content and tenderness. In addition, cooking loss in US-cooked meat is 2–5 times less than meat cooked by boiling and convection. This indicates that the possible uses of US are important in providing pre-cooked or cooked meats for use in prepared meals in the food processing or food service industries [27]. US cooking has also been applied on mortadellas [44], spiced beef [202], [203], other red and poultry meats [27], [4], [6], [89], crab [37], [38], corn [82], [83], [123], [149], and pasta [146].

Incidentally, some recent studies related to US cooking have shown that this method affects the color properties of products. da Silva et al. [44] examined the reduction in cooking time of mortadella using US and reported no significant differences in the color of the US-cooked mortadella. In the study, a*/b* values of the US-cooked samples were similar to the control sample (p > 0.05), but cooked samples (without US) had lower a*/b* values (p < 0.01). These results suggest that the oxidation of nitrosyl hemochrome pigment (pink color) is not assisted with US cooking. ΔE values of the samples cooked by US were found between 1.49 and 1.53. It was shown that the color differences due to the US application might not be discerned by the consumers when the ΔE values were below 2. In another study, the effect of US on the technological properties of chickpea cooking water (aquafaba) was investigated where the high-intensity US was applied for 30 min [119]. It was seen that US treatment increased the stability, texture, and color of foams. Moreover, the French meringue prepared using treated aquafaba had better texture and color than the meringue made with untreated aquafaba. The L* values of the treated foams with US were significantly higher than the untreated sample (p < 0.05). These results were similar to the L* values obtained from the meringue made using treated aquafaba. On the other hand, an opposite situation was observed in ΔE values for meringue made with treated aquafaba (p < 0.05). Some research has also been carried out on the utilization of US for cooking meats. The results obtained from these studies have indicated that US-assisted cooking may be an effective technique to improve the quality and chemical profiles of spiced beef flavor and taste [202], [203]. Alarcon-Rojo et al. [5] conducted a study on US-assisted cooking and noted that color changes in meat due to exposure to the high-intensity US might not be significant for treated or cooked beef or other meat species such as rabbit, pork, and chicken. In addition, the application of high-intensity US can be seen as a promising technology for cooked beef, pork, and rabbit as it does not adversely impact the color. Some studies also support these results, as they suggest that the heat produced is not enough to denature pigments and proteins, and therefore US does not affect the meat color [164].

In addition to meats, US cooking is also used for cereal products. Advances in genetics and omics technologies enabled the improvement of the quality and the nutritional value of cereals [69], applied genetics being used for enhancing the nutritional quality, especially in improved colored cereals, by targeting specific genes engineered for improving or reducing the value of key nutritional compounds [41]. Apart from production technologies, the processing technologies such as US also play a major role in maintain the color profile of food. For this purpose, Raso Pueyo and Álvarez Lanzarote [146] employed the US method on different products during the cooking process and found that US-assisted cooking of pasta at boiling temperatures reduces the optimal cooking time by 20 % compared to traditional cooking. This was assumed to be achieved as a result of US facilitating the gelatinization of starch without affecting the resistant starch content and thereby improving the color and stickiness of the product. The optimum cooking time was determined as 10 min for control cooking and 8 min for US-assisted cooking. These times did not make a significant difference in L* and a* values but made a significant difference for b* value. US cooking better preserved the yellow color in the final product, indicating less pigment loss, possibly due to the shorter exposure of the pasta to the cooking water at 100 °C. As a result, the researchers stated that US-assisted cooking did not adversely affect other sensory properties. On the contrary, it reduced the cooking time and helped preserve the characteristic light-yellow color of the pasta. US-assisted cooking has been successfully employed to enhance the nixtamalization of corns. Nixtamalization is a crucial production stage for masa flour used for making corn tortillas, tacos, and chips. This process traditionally includes cooking the corn kernels for 1 h and a long soaking process (16–18 h) in a lime solution. Janve et al. [82] aimed to accelerate the traditional nixtamalization process in their study. For this purpose, the researchers used US power for 1 h during cooking followed by short steeping for 1 h. The US treatment significantly differed in the samples’ L*, C*, and ΔE values (p < 0.05). This was related to the cavitation effect of the US being adequate. It was stated that the overall color properties of treated samples with US were better than the traditionally nixtamalized samples. In another study on maize, Moreno-Castro et al. [123] applied US during nixtamalization. The process was carried out at different temperatures (85 °C and 95 °C). The maize treated with US at 95 °C had the highest L* value, whereas the lowest L* value was determined in the sample obtained at 95 °C without US (p < 0.05). These values were significantly different from the samples obtained at 85 °C with and without US (p < 0.05). There was no significant difference between the b* values of the samples obtained at 85 °C and 95 °C without US (p > 0.05). However, the yellow color of the maize treated with US at 95 °C was more intense than the others. The a* values of the maize showed significant differences only at 95 °C (p < 0.05), and the highest value was determined in the sample obtained at 95 °C without US. No statistically significant difference was observed between L* and b* values of the tortillas obtained from all treatments (p > 0.05). Contrary to the values obtained for maize, there was a significant difference in the a* values of the tortillas obtained only at 85 °C (p < 0.05), and the application of US decreased the redness of these tortillas.

Similarly, Robles-Ozuna et al. [149] applied US to the corn during the nixtamalization process, and then this sample was used for the production of pozole. A lower b* value was observed when US was applied during the process. The amount of lime retained during the washing of the nixtamal (cooked corn) may affect the color changes in the nixtamalized grain. The reactions between the lime and the natural pigments (carotenoids) in the maize cause yellow and dark coloration. However, removing a considerable amount of the pericarp during cooking may affect this situation [152]. This is more evident during the US application because removing the pericarp is more apparent [123]. There were significant changes in the h°, C*, L*, and a* values when the nixtamalization of the corn was assisted by US (p < 0.05). The b* values of the corns nixtamalized by US ranged from 18.2 to 19.5, and a significant difference was not found between them (p > 0.05) in cooking time. The L* values of the US- nixtamalized samples were comparatively higher than the values observed in the corns that were traditionally nixtamalized. This situation may be related to the US applied to the cooking water in the dehulling of the pericarp. The application increased the water absorption of the grain [123] and made a better distribution among the grain components. This can lead to higher brightness in the corns compared to the traditional method. In general, the study demonstrated that traditional and US-assisted nixtamalization changed the color parameters, but the US treatment was more advantageous, and the values obtained from the US-nixtamalized samples were more coherent.

In a study that set out to compare the properties of traditional with US-assisted processed corns, Janve et al. [83] found that the L* value of the masa flour treated with US was higher, and the final color of the masa was significantly lighter (p < 0.05). The ΔE value of the nixtamalized maize by US method was significantly lower than that by the traditional method, whereas h° was significantly higher (p < 0.05). However, the ΔE value has to be above 3 to be visible by the human eye [83]. Traditional and US processes made no significant difference between the C* values of the maize (p > 0.05). The researchers reported no significant difference in the ΔE and C* values of the baked chips prepared with maize flours treated with both the processes (p > 0.05). The baked chips made with nixtamalized maize flour by US had lighter color and, therefore, higher L* values (p < 0.05). Higher h° values were obtained in these samples (p < 0.05). The tortilla chips made with flours prepared from US-nixtamalized corn showed significant differences in lower ΔE values and higher L* and h° values (p < 0.05). However, there was no significant effect on the C* values due to the methods applied. In conclusion, the application of US in the production of tortilla chips caused substantial differences between the masa flours (raw material) (L*, ΔE, and h°), the baked chips (intermediate product) (L* and h°), and the tortilla chips (final product) (L*, ΔE, and h°) in terms of color characteristics. Although the two methods did not make differences between the water absorption values of the samples, higher lime concentration or shorter steeping time in the US method may have caused changes in color components.

Therefore, it can be seen that US process, when used as an assistance for cooking, has proven to reduce the cooking time and simultaneously improve the color properties of different food products. The color differences between the treated and fresh samples were found insignificant in most cases, which shows the capability of ultrasound in positively influencing the cooking process once the parameters are optimized.

### 3.5 Effect of ultrasonic drying

Fresh foods often have a high moisture content (such as fruit, vegetables, and animal products). While the high moisture content of these foods is primarily open to microbial spoilage, it can also bring chemical spoilage. For this reason, one of the preferred ways of preserving food is to remove water from the structure. Many methods are used for this purpose in the food industry. Among these methods, while sun drying continues to exist as a traditional method used since ancient times, different techniques are being explored because sun-drying could only take place in more than a few days, and the products obtained cannot meet international standards [153].

Among these methods, ultrasonication is a preferred process lately because it does not increase the product temperature, accelerates heat and mass transfer, and is cost-efficient [79]. Unlike other methods, there is no liquid phase change with ultrasonic drying application, and it is one of the novel methods that appears as a supportive process in the food industry since it is not possible to remove water completely. The use of ultrasonic waves for drying foods dates back to the 1950s [125]. While low-intensity ultrasonic waves can be used to determine some structural features in foods, high-intensity ultrasonic waves (10–10000 W/cm^2^) can be applied in much wider power ranges and are used in situations where changes are desirable in the material such as drying process [11]. High-intensity ultrasonic waves cause contraction and relaxation in the material they come into contact with. The resulting stress accelerates the movement of water with the help of microchannels. In addition, the cavitation caused by ultrasonic waves is also very effective in water movement. The US can be used as a supporting method and a pre-treatment in the drying of foods. Ultrasonic applications are effective in removing water by reducing the resistance between the structure and the environment in different environments, such as solid–liquid, and solid–gas, as well as contributing to drying by reducing the internal resistance as a pre-treatment [147]. Ultrasonication is preferred pre-drying pre-treatments to prevent enzymatic and non-enzymatic browning changes that affect color properties in foods. In the studies on fruits and vegetables where US application is widely used for drying. It was stated that the mean ΔE* decreased as US power progressed from 0 to 1000 W, and a similar correlation was obtained between US power and drying time. The reason for this color change is explained by ultrasonication restriction of enzymatic oxidation reactions. Similarly, there are studies where US applications delay these enzymatic browning reactions, limit enzymatic activities such as polyphenol oxidase and peroxidase, and the color properties of the product can be preserved for a longer time [148], [112], [98]. While US applications contribute to preserving color pigments such as carotenoids in the drying process [42], there are also negative effects reported in the literature. Such as, US caused heat-resistant green and yellow pigment losses in green beans [175], carotenoid losses in melons [43], color changes in tomato slices [72], and color changes of lotus flower seeds [200]. The US process can remove gases from the structure as well as shorten the drying time and limit the retention of pigments in the structure and color change reactions [48].

Most studies concur that high intensity promotes oxidation of both lipid and protein components when assessing oxidative stability. Off-flavors and off-odors are generally produced by lipid oxidation, which has a negative impact on sensory qualities [5]. For this reason, US-assisted drying studies in fish, meat, and meat products are very limited in the literature. To give insight into these studies, Kadam et al. [87] stated that US pretreatment increased the L* value and b* value of the product while decreasing the a* value compared to the control group in hot air drying seaweeds. Li et al. [104] determined the whiteness value to evaluate the color change where they used US-assisted osmosis pretreatment in trehalose solution for drying tilapia fillets with a heat pump. In the study, ultrasonication application maximized the whiteness value up to 60 min, and still higher whiteness values were achieved after 60 min compared to the non-applied process. It has been stated that this increase in whiteness value is due to the spongy structure formed due to the rise in microchannels formed by ultrasonication and that it can come into contact with the osmotic liquid at a high rate with the increased surface area. In the study of Aksoy et al. [3], it was stated that the minced meat color is preserved for a longer time with US and vacuum-assisted drying processes in the drying of minced meat and US application at 45 °C. With US-assisted vacuum drying, shorter drying time, less color loss, and lower shrinkage were detected compared to vacuum drying.

In short, US-assisted drying has shown both beneficial and detrimental effects on the color properties of different foods; however, with the impending application for inactivating enzymes responsible for browning. Also, by disintegrating the cells, ultrasound has been shown to homogenize the product yielding a brighter color in the final product.

### 3.6 Effect of ultrasonic pasteurization

Thermal pasteurization is a gentle heat treatment that is applied to foods with the goal of destroying the target vegetative cells. Pasteurized foods are not shelf-stable and must be kept refrigerated or packaged in a modified atmosphere. Pasteurized goods can last anywhere from a few days (milk) to many weeks (fruit juices). The nature of the product, pH, heat resistance of the target microorganisms, susceptibility of the product quality to heat damage, and the heating technique determine the severity of heat treatment utilized (time and temperature) and the length of shelf-life attained [174]. While heat treatments are successful in inactivating bacteria, enzymatic activity, lipid oxidation, and protein denaturation, they have a detrimental impact on foods due to vitamin losses and changes in sensory qualities such as color, taste, and bioactivity [33]. For this reason, non-thermal processes are preferred instead of these thermal processes, which aim to preserve the nutritional properties of the food, provide lower energy consumption, and be efficient [122].

One of the non-thermal processes is US application. In liquids, US generates areas of extremely high temperature and pressure peaks. The quantity and intensity of bubble implosions per unit of time, the therapy, and the features of the treatment media all influence the effects of high-intensity US. When the ultrasonic energy supplied is insufficient to keep the vapor phase in the bubble, fast condensation ensues. Shock waves are created when condensed molecules clash forcefully. These shock waves generate temperatures and pressures of up to 5500 °C [68]. It is stated that inhibition can be achieved in the number of microorganisms due to reasons such as damage to the cell wall of microorganisms and heat change associated with temperature change and cavitation effect provided by US. It is more common to use US with non-heat applications and various antimicrobial agents [201]. The impact of US on microorganisms varies depending on the target microorganism, the physical and chemical properties of the applied food, the processing time, and the power used [66].

Ultrasound is widely applied to pasteurize liquid foods such as pomegranate juice, gooseberry juice, etc., where different color properties have also been evaluated [16], [132]. Color changes due to thermal treatment can be explained due to the non-enzymatic browning and destruction of color pigments, while US can cause carotenoid isomerization and degradation due to bubble formation, collapse, and other sonochemical reactions. However, US can also interact with the Maillard reaction, thereby retarding the generation of darker pigments and preserving the luminosity of the sample [18], [155], [52].

Ultrasonication can increase turbidity values by the suspension of microorganisms and small molecules that can be released as a result of the destruction of large molecules as a result of cavitation during sonication, and that the free radicals formed may come together with phenolic components and support the formation of water-insoluble polyphenols [52], [50], [184].

Decreased L* and b* values were detected in almond milk [117] and hazelnut milk [13] by thermosonication (TS), a process in which ultrasound and thermal process are used together. TS process applied above 50 °C can trigger the Maillard reaction [117], [13]. A similar application obtained higher color density, L*, and ΔE values with the TS process applied to blueberry juice [187].

While there are many studies in which US application is effective on microbial inhibition in liquid foods [122], [155], these studies on solid foods are very limited. Sert et al. [158] exposed the shelled egg to US for 5, 15, and 30 min using an ultrasonic bath with a power of 35 kHz-140 W. After the procedure, a* values of yolk increased, and L* and b* values of albumen decreased. Ultrasonic treatment reduced water loss, inhibited microbial growth, and improved the quality of egg yolk and albumen. In another study, it was reported that high-pressure CO_2_ application and high-power US application did not affect the color properties of the product (L*, a*, and b*) in the dry-cured raw product [156]. Ferrentino and Spilimbergo [58] stated that US significantly impacts microorganism reduction by enhancing the contact of pressurized CO_2_ with the cells' surfaces. It has been reported that pasteurization does not cause significant differences in L*, a*, and b* values of carrots. Cichoski et al. [34] also detected a similar effect in hot dog sausages. While L* and b* values of sausages were not affected in a TS-applied process, a* value decreased [77].

### 3.7 Other applications

Ultrasonication has easily adapted to the food industry thanks to its many superior features. In addition to its common usage areas (cooking, drying, extraction, pasteurization, thawing, irradiation, etc.). The US has also found other miscellaneous applications [115].

One of them is the frying process. Frying is a widespread practice in preparing foods, such as deep fat frying. However, during this application, there may be cooking differences between the interior and exterior parts due to the high-temperature effect, depending on the size and characteristics of the product. With US application, US support can be applied in the frying process with the effect of pressure and temperature changes occurring in the cellular dimension during cavitation. Ultrasound can increase heat transfer by convection and conduction between product and oil. Wang et al. [181] examined increased L*, a*, and b* values of US-supported fried meatballs. However, it was stated that the US pretreatment applied to fry the potato did not affect the color properties [170]. Studies in which US application does not affect color properties in frying are quite common [73], [49]. It is mentioned that US is an application that can be preferred in the frying process in terms of product color properties.

Another practice is blanching, or scalding as it is sometimes called, which is a key step in the preparation of vegetables for freezing, canning, or dehydrating. Blanching aims to remove the gas from the product structure, reduce the volume for adequate filling, and preheat if the canning process is to be used in the storage of vegetables while inactivating the enzymes before the freezing process to prevent spoilage that may be caused during storage [102]. US waves can be effective in the inactivation of many enzymes due to the cavitation they provide, and the fact that they are very effective in transferring heat shows that they can be a non-thermal method for blanching pretreatment [189]. Lespinard et al. [103] stated that the loss of L* value from color properties decreased and polyphenol oxidase enzyme inactivation accelerated by the support of 60, 70, 80, and 90 °C temperature applications with US treatment in the blanching process of mushrooms. During blanching, decreasing L* value indicates that the samples get darkened. This darkening could be ascribed to the disruption to the cellular membrane of mushrooms, encouraging polyphenol oxidase (PPO) contact with its substrate and resulting in the sample browning. Another study determined the effectiveness of hot water, US, and thermosonication processes in the blanching application of carrot slices. US (dual and tri-frequency) application alone was insufficient to inhibit peroxidase enzyme activity and did not cause a significant difference in L*, a*, and b* values [165].

Bleaching is a widespread procedure in the oil business for removing pigments and decolorizing oil. For this purpose, typically heat bleaching, chemical oxidation, and adsorption methods are used. With the use of US in the bleaching process of rapeseed oil, the carotene is broken down (A446) without the need for adsorbent [169]. In a similar study, an optimization study was carried out with 400 W, 24 kHz US support in the bleaching process of soybean structure. As a result of the optimization, it was determined that while the red and yellow color indicators decreased with the US process, almost all carotenoid and chlorophyll were removed. With the study, it has been determined that US support can provide lower clay usage, lower temperature, and a shorter bleaching process [1]. Similar results have been reported with the use of canola oil [76].

Using an ultrasonic knife for cutting or size reduction is a new method that is gaining traction in the food industry. The ultrasonic frequency of reciprocal vibration of a cutting knife considerably lowers friction between the blade and the product, resulting in clean cuts and products of consistent size, shape, and density [56]. Yildiz et al. [192] reported that US application increased the L* value, 50% amplitude US application gave the maximum L* value, and the L* value of all samples decreased during storage as a result of the ultrasonic cutting process applied in two different apple species, a*, and b* reported an increase in values. Polyphenol oxidase activity causes enzymatic browning reactions in fruit. Since the cells are less damaged after ultrasonic cutting, their contact with oxygen is less than in control samples. As a result, the polyphenol oxidase enzyme activity could explain the low color changes, which decreased in line with the US intensity. In another study, while ultrasonic cutting of cheddar, mozzarella, and Swiss cheeses provided high L* values no significant changes were found in a* and b* values [191].

## 4 Challenges and future perspectives

With numerous advantages in the food industry pertaining to the nutritional and sensory aspects of food, ultrasound processing or pretreatment possess certain limitations and challenges which is to be dealt with. Firstly, the different free radicals formed during the cavitation process due to high temperature and pressure can trigger the stimulation of radical chain reactions forming many degradation products that can cause harmful effects on the consumer [20]. Also, when used at higher intensities, considerable heat gets generated, which can cause detrimental effects on the organoleptic and nutritional characteristics of foods. The other important sonochemical reactions associated with ultrasound processing include micro‐scale bubble collapse, degassing, improved diffusion, and enhanced polymerization/depolymerization reactions, which could either occur simultaneously or separately [124]. Such reactions cause can cause chemical decomposition of color pigments affecting the appearance of the final processed products. With the uncontrolled rise in temperatures and pressures, a phenomenon called the ‘cushioning effect’ happens, which minimizes the collapse of cavitation bubbles that can cause ring-opening and other changes in the pigments leading to degradation. Subsequently, accelerated isomerization of color pigments can also occur, which is a major challenge associated with ultrasound processing.

The degradation products and their by-products differ with different products, demanding other optimal processing parameters since the temperature rise during sonication can result in thermal degradation of these pigments [124]. Consequently, further research is still essential for specific sonication applications since different factors have to be considered while designing the equipment and optimizing the operating parameters for efficient application as far as color parameters and other sensory properties are considered. The input energy demand for ultrasonication is sufficiently high, which mandates proper industrial intervention for the commercialization of this technique. Also, with more significant changes in the final product quality with increasing ultrasonic power, minimizing power is an ensuing task to be given importance for future research works for achieving proposed results.

## 5 Conclusions

Food color plays one of the most critical roles in deciding the consumer perception of processed or unprocessed food. There are dominant pigments that determine the color of each food; the most important pigments are anthocyanins (red–purple color), chlorophylls (green color), carotenoids (yellow-orange color), and betalains (red color). Pigments present in foods, especially in fruits and vegetables, belong to diverse groups of chemical substances that differ in color, stability, solubility, and sensitivity to environmental conditions in the presence of other substances, and they can be lost during food processing. The present review collates information on various aspects of ultrasonication technology, its mechanism of action, influencing factors, and the competence of different ultrasonication applications (drying, irradiation, extraction, pasteurization, cooking, tempering, etc.) in preserving the color of food. In conclusion, it has been seen that the effects of ultrasonication applications on the colors of foods are quite positive. The proper determination of the ultrasonication conditions used is very effective in preserving the color of the food. For this purpose, since the processing conditions of each food will differ, the importance of optimization studies for ultrasonic applications is obvious, so ultrasonication applications on food colors are still open to study. In addition, minimizing the energy consumption and the power while preserving the color of the foods is one of the subjects available to research in this field. As future research recommendations, optimizing process conditions and energy/exergy analysis of US applications can be recommended for researchers.

## CRediT authorship contribution statement

**Naciye Kutlu:** Conceptualization, Writing – original draft, Writing – review & editing. **R. Pandiselvam:** Conceptualization, Resources, Writing – original draft, Writing – review & editing. **Aybike Kamiloglu:** Writing – original draft. **Irem Saka:** Writing – original draft. **N.U. Sruthi:** Writing – original draft. **Anjineyulu Kothakota:** Writing – original draft. **Claudia Terezia Socol:** Resources, Writing – review & editing. **Cristina Maria Maerescu:** Conceptualization, Writing – review & editing.

## Declaration of Competing Interest

The authors declare that they have no known competing financial interests or personal relationships that could have appeared to influence the work reported in this paper.

## Data Availability

Data will be made available on request.
